# Understanding the Physiological Links Between Physical Frailty and Cognitive Decline

**DOI:** 10.14336/AD.2019.0521

**Published:** 2020-03-09

**Authors:** Lina Ma, Piu Chan

**Affiliations:** ^1^Department of Geriatrics, Xuanwu Hospital, Capital Medical University, Beijing Institute of Geriatrics, Beijing, China; ^2^China National Clinical Research Center for Geriatric Medicine, Beijing, China; ^3^Department of Neurology and Neurobiology, Xuanwu Hospital, Capital Medical University, Beijing, China; ^4^Key Laboratory for Neurodegenerative Disease of the Ministry of Education, Beijing Key Laboratory for Parkinson’s Disease, Parkinson Disease Center of Beijing Institute for Brain Disorders, Beijing, China

**Keywords:** physical frailty, cognitive decline, biology, cognitive frailty

## Abstract

Declines in both physical and cognitive function are associated with increasing age. Understanding the physiological link between physical frailty and cognitive decline may allow us to develop interventions that prevent and treat both conditions. Although there is significant epidemiological evidence linking physical frailty to cognitive decline, a complete understanding of the underpinning biological basis of the two disorders remains fragmented. This narrative review discusses insights into the potential roles of chronic inflammation, impaired hypothalamic-pituitary axis stress response, imbalanced energy metabolism, mitochondrial dysfunction, oxidative stress, and neuroendocrine dysfunction linking physical frailty with cognitive decline. We highlight the importance of easier identification of strategic approaches delaying the progression and onset of physical frailty and cognitive decline as well as preventing disability in the older population.

Declines in both physical and cognitive function are associated with increasing age. Frailty is characterized by failure of homeostatic mechanisms and vulnerability to adverse outcomes [1]. The prevalence of frailty is 3.5-51.4% across different geographical regions [2-6]. However, there is no consensus regarding the single definition of frailty for clinical application. There are two major operational definitions for frailty. The most widely used concept is the Fried physical frailty phenotype, which defines frailty based on three or more of the following five symptoms: unintentional weight loss, slowness, weakness, exhaustion, and low physical activity [7]. Sarcopenia, a condition of loss of muscle mass and function, increases the risk of physical frailty and is associated with cognitive impairment [8]. The second widely used concept is Rockwood frailty index composes many clinical conditions and diseases[9] and is a marker of deficits accumulation based on comprehensive geriatric assessment [10]. Both physical frailty and frailty index are associated with late-life cognitive impairment [11,12].

Cognitive frailty was defined as the simultaneous presence of physical frailty operationalized based on the Fried phenotypic model and mild cognitive impairment (MCI) without dementia by an international consensus group from the International Academy of Nutrition and Aging (IANA) and the International Association of Gerontology and Geriatrics (IAGG) [13]. Recently, two subtypes of the new construct were proposed: reversible cognitive frailty and potentially reversible cognitive frailty [14]. An updated version of cognitive frailty model is presented in Figure 1. The prevalence of cognitive frailty ranges from 10.7% to 22.0% in clinical-based setting and from 1.0% to 4.4% in population-based setting [15]. Cognitive frailty is associated with increased risk of functional disability, poor quality of life, and mortality.

**Figure 1.**
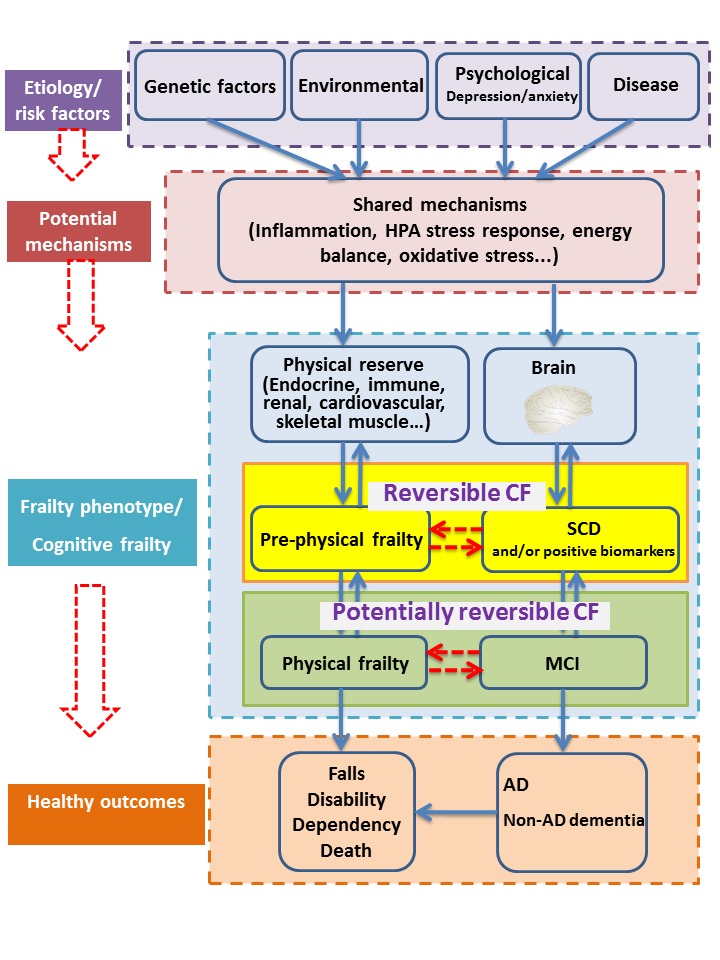
The model of cognitive frailty. Physical frailty and cognitive impairment have the same etiology, and might share the same mechanisms, which lead to adverse health outcomes. The decline in physical reserve and cognitive function contribute to frailty and cognitive impairment separately. Cognitive frailty is the combination of frailty and cognitive impairment in absence of dementia, which is further divided into reversible cognitive frailty (prefrailty and subjective cognitive decline) and potentially reversible cognitive frailty (physical frailty and mild cognitive impairment).Abbreviations: CF, cognitive frailty; SCD, subjective cognitive decline; MCI, mild cognitive impairment; AD, Alzheimer's disease.

Understanding the physiological link between physical frailty and cognitive decline may allow us to develop interventions that prevent and treat both conditions and thus, improve independent function and quality of life in older individuals. Although there is significant epidemiological evidence linking physical frailty to cognitive decline [11,12,16], a complete understanding of the underpinning biological basis of the two conditions remains fragmented. The mechanisms underlying cognitive-frailty link are multifactorial since inflammatory, nutritional, vascular, and metabolic factors may be involved [17]. Sarcopenia may also explain this link [15]. Aging is associated with immunosenescence, which is characterized by declines in adaptive and innate immunity [18]. The central nervous system and the immune system are constantly interacting [19]. In addition, impaired hypothalamic-pituitary axis (HPA) stress responses, imbalanced energy metabolism, mitochondrial dysfunction, oxidative stress, and neuroendocrine dysfunction may be associated with both physical and cognitive decline, and thus may be involved in mechanisms underlying the link between physical frailty and cognitive decline (Fig. 2).

**Figure 2.**
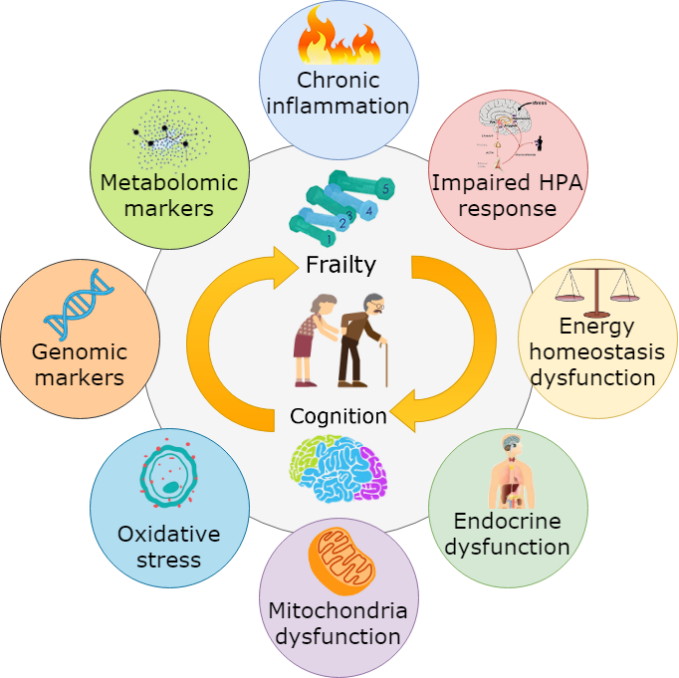
Overview of the underlying mechanisms linking physical frailty to cognitive decline. Chronic inflammation, impaired HPA stress response, imbalanced energy metabolism, endocrine dysregulation, mitochondrial dysfunction, oxidative stress, genomic markers and metabolomic markers are major underlying mechanisms between physical frailty (muscle) and cognitive decline (brain).

## Chronic inflammation

Inflammaging refers to the low-grade systemic pro-inflammatory state resulting from the upregulation of the inflammatory response driven by multiple factors in old age [20,21]. It is characterized by high susceptibility to morbidity, frailty, disability, and mortality [22]. Chronic inflammation is associated with poor physical performance [23]. Pro-inflammatory cytokines include interleukin 6 (IL-6), IL-1β, IL-12, and tissue necrosis factor alpha (TNF-α) as well as C-reactive protein (CRP). Chronic inflammation contributes to the increased risk of frailty, potentially mediated via neurodegeneration [24]. TNF-α and IL-6 influence the onset of frailty and cognitive decline [25], and CRP levels link muscle quality with cognitive function [26]. Anti-inflammatory cytokines include IL-10, IL-4, IL-13, and IL-1Ra [27]. The deregulated balance between the pro- and anti-inflammatory status may induce lower physical function, thus affecting the central nervous system, and is involved in the pathophysiological mechanisms of frailty and dementia.

Frailty is associated with chronic inflammation [28]. High levels of IL-6, TNF-α, and CRP were found to be associated with poor function and mobility status [29,30], lower muscle strength and muscle mass, and frailty in older individuals [31-35]. Systemic inflammation enhanced inflammatory responses within the central nervous system, contributing to cognitive decline [36,37]. Peripheral cytokines showed a direct influence on the central nervous system [19]. High levels of IL-1, IL-6, CRP, and TNF-α were also found to be potentially predictive markers for the development of Alzheimer's disease (AD) or cognitive decline [38-42]. Some studies showed that high levels of CRP were found in senile plaques and neurofibrillary tangles in the brain of AD patients [43,44]. However, other studies have failed to show the relationship between chronic inflammation and cognitive decline [45,46].

IL-6 is the most important cytokine in inflammaging. Serum IL-6 levels increase with age, independent of other comorbid disease processes [47,48], and are associated with poor physical performance (slower gait velocity and muscle weakness) and worse cognitive function. Rise in serum IL-6 levels are predictive of poor physical and cognitive performance, disability, and mortality in the older population [30,34,49-55]. IL-6 and the IL-6 receptor (IL-6R) promote chronic inflammation in the central nervous system and contribute to the development of AD [56]. Higher IL-6 levels are associated with muscle atrophy [57] and global and hippocampal atrophy [58], and may account for the association between AD pathology and frailty, independent of a dementia diagnosis [59]. TNF-α and its soluble receptor had the strongest association with muscle mass and strength decline in older persons [60]. Moreover, they were associated with both functional and cognitive decline [61]. Elevated TNF-α levels in the cerebrospinal fluid (CSF)[62], serum [63], and brain [64] have been observed in AD patients. High plasma TNF-α levels are predictive of muscle strength and cognitive declines [60,65]. Soluble TNF receptor 1 (sTNFR1) can differentiate between MCI and AD and may be helpful in determining the degree of cognitive impairment [66]. As an anti-inflammatory cytokine, lack of IL-10 leads to increased expression of nuclear factor-κB (NF-κB)-induced inflammatory mediators [67], reduced skeletal muscle energy metabolism, and reduced release of free energy [68]. Moreover, IL-10 was negatively associated with executive function and processing speed. Higher neutrophil and monocyte counts, as well as lower lymphocyte counts, were associated with low physical activity [69] and frailty [70]. Elevated fibrinogen levels were associated with frailty[28], and decline in cognition [71,72], and predicted the onset of cognition deficits [46].

Recently, multivariable measures of inflammation provided an easier approach to track the progression of frailty over time. For instance, the inflammatory index score based on IL-6 and sTNFR1 has been shown to best describe age-associated chronic inflammation as well as predict mortality; moreover, the score was higher in frail older adults than in robust participants [73]. An index based on seven circulating inflammatory molecules was independently associated with deteriorating mobility function and frailty risk [74]. The above indexes were not studied in cognition.

## Hypothalamic-pituitary axis stress response dysfunction

The HPA axis dysfunction is a pathway that contributes to both physical frailty and cognitive decline. The levels of dehydroepiandrosterone sulfate (DHEA-S), testosterone and growth hormone (GH) decrease, while cortisol levels increase with age [75-77]. Multiple hormonal changes play a major role in the development of frailty, sarcopenia, cognitive decline and mortality in older adults [77,78].

Cortisol, a lipophilic steroid hormone produced in the cortex of the adrenal glands, contributes to vulnerability to stressors in frail patients. Frail older adults display higher levels and blunted diurnal variation of cortisol [76,79,80]. Increased basal cortisol levels contribute to cognitive decline and may be associated with decreased hippocampal volume in AD patients [81]. Higher levels of cortisol were associated with lower brain volume and impaired memory in asymptomatic younger to middle-aged adults [82] and worse performance in cognitive domains in adults aged 50 to 70 years [81].

Reduced testosterone levels may mediate the relationship between physical frailty and cognitive decline. Age-related depletion of testosterone was associated with muscle mass decline [83]. Grip strength and physical activity were associated with total testosterone levels [84]. Testosterone had protective effects on cognition by promoting synaptic plasticity in the hippocampus and regulating the accumulation of Aβ protein [77]. Reduced androgen hormone levels may be related with both frailty and cognitive decline, and some hormonal changes have been shown to directly influence skeletal muscle decline and cognition [77,83]. DHEA-S was also lower in frail people [85].

GH levels decrease with age and are related to both frailty and cognitive impairment [86]. Learning and memory are induced by GH, and GH therapy could improve cognition, especially in behavioral disorders of the central nervous system [87]. GH-releasing hormone therapy has a positive effect on cognition in MCI participants [88].

## Energy homeostasis dysfunction

Energy homeostasis dysfunction may provide another link between physical frailty and cognition. Decreased serum levels of the anabolic hormone insulin-like growth factor -1 (IGF-1), were found in both frail older adults [89,90] and AD patients [91]. Elevated serum IGF-1 levels are positively correlated with physical performance [92], thigh muscle area and density [93], knee extensor strength, and difficulty in mobility-related tasks [94], and negatively associated with muscle cell apoptosis [95] and poor health outcomes [96]. Both the secretion and biological actions of IGF-1 are modulated by pro-inflammatory cytokines. The negative effect of IL-6 on muscle function is exerted through IGF-1[97], while the effect of IGF-1 on muscle function depends on IL-6 levels [98]. A pro-inflammatory state had a significant detrimental effect on frailty; only under normal endocrine function, in cognitively impaired older adults [24]. Hence, the combined influence in frailty and cognitive decline requires greater in-depth exploration.

Silent mating-type information regulation 2 homolog 1 (SIRT1) is a key regulator of aging-related metabolic changes. Serum SIRT1 levels declined with age [99], and low SIRT1 levels were found in both patients with AD and MCI [100] and frail participants [101]. Other studies showed that increased SIRT1 activity was associated with both delayed aging [102] and cognitive decline [103]. There is a paradoxical association between low serum SIRT1 levels and robustness [104]. A recent study showed that higher serum SIRT1 levels in frail older adults were associated with slow walking speed [105]. SIRT1 single-nucleotide polymorphisms (SNPs) and serum SIRT1 levels in older men were possibly more closely associated with nutrition and body composition than with aging and age-related conditions [106]. Another study found no association between frailty and serum SIRT1 levels [104]. Ghrelin contributes significantly to the development of both physical frailty and cognitive impairment by stimulating gastric acid secretion, regulating glucose metabolism and energy homeostasis, and improving learning and memory [107]. Frail women had lower levels of fasting and 120 min ghrelin [108]. Ghrelin deletion prevented the decline in muscle strength and endurance by attenuating the decrease in phosphorylated adenosine monophosphate-activated protein kinase and increasing the number of type IIa muscle fibers [109]. Ghrelin was also involved in the neuro-modulation, neuro-protection and memory and learning processes [110]. Reduced ghrelin levels were associated with MCI in type 2 diabetes (T2DM) populations [111] and with metabolic changes in AD patients [112]. However, a recent study found that ghrelin modulated encoding-related brain function without enhancing memory formation in humans [113].

## Endocrine dysregulation

Endocrine dysregulation is involved in the progression of physical frailty and cognitive decline by accelerating immunosenescence, attenuating neuroprotective and neurotrophic effects, and promoting muscle catabolism [114]. Clegg reviewed evidence on the association between frailty and the endocrine system [115]. However, the role of endocrine alterations in the etiology of frailty and cognitive decline is still poorly understood. Circulating adiponectin and leptin have been interrogated in many studies with conflicting results.

Insulin resistance (IR) was associated with incident frailty and poor cognitive function [116-119]. Higher Homoeostatic Model Assessment for IR index values were associated with a higher risk of frailty[120]. AD is considered as type 3 diabetes mellitus [121]; IR is an important risk factor for cognitive impairment in older adults [119,122]. Furthermore, rosiglitazone could improve learning and memory ability by normalizing the impaired insulin signaling pathway in diabetic rats [123]. Vaspin is a visceral adipose tissue-derived serine protease inhibitor with insulin-sensitizing effects associated with IR. Circulating vaspin levels increased with aging and were associated with parasympathetic activity even in the absence of metabolic syndrome [124]. Frail older adults showed higher levels of vaspin compared to participants who did not show frailty [125].

Adiponectin is a pleiotropic adipokine inversely correlated with adipose tissue dysfunction. Epidemiological findings indicate a paradoxical involvement of adiponectin in the health status. High levels of adiponectin were associated with decreased muscle strength [126], grip strength [125], frailty [125], increased number of frailty components [127], higher incidence of cardiovascular diseases and disability, and high mortality rate [128], but a low risk of T2DM [129]. Adiponectin levels have been associated with MCI and AD, while higher plasma adiponectin was associated with poor cognitive performance, neuroimaging and cognitive outcomes in women [130]; in addition, another study showed that serum adiponectin was positively associated with better cognition in the postmenopausal period [131]. The adipocyte-derived hormone leptin regulates body weight and metabolism. Its secretion links food intake and energy reserves with energy expenditure, growth, and reproduction. Higher leptin levels were associated with higher risk of frailty, which was modestly explained by IR and chronic inflammation [132]. Circulating leptin was inversely correlated with gait speed [125]. Increasing leptin levels with increasing muscle mass showed positive effects on the skeleton mas s[133]. Leptin receptors impact cognitive function by affecting hippocampal synaptic plasticity[134]. Leptin resistance was linked with the development of AD [135]. High levels of leptin were associated with improved cognition in T2DM patients [136], while other studies found no association with function or global cognition [61], and blood leptin levels were not correlated with cognition in AD patients [137].

## Mitochondrial dysfunction

Mitochondria contribute to the dynamics of cellular metabolism and reactive oxygen species (ROS) production. Thus, their role in aging has drawn much attention over the years. Increased levels of free radicals activate the NF-κB pathway. Mitochondrial function has been associated with physical function and vulnerability to disease in older adults [138-140]. The accumulation of mitochondrial and nuclear DNA damage leads to the loss of myocytes and muscle wasting [141]. Recent studies found that improving mitochondrial function reduced metabolic, visual, motor, and cognitive decline in aged *Drosophila melanogaster* [142].

Mitochondria are important sources of endogenous damage-associated molecular patterns and activate an innate immune response [143,144]. Mitochondrial DNA (mtDNA) is a known surrogate marker of whole-body mitochondrial function [145]. Low mtDNA levels were associated with frailty, poor physical strength and mortality, while high mtDNA levels were associated with better health and longevity [138,139]. Some studies have found that mtDNA levels in plasma increased with age [146]. Increased plasma mtDNA is a marker of ongoing inflammation and better neurocognitive function in virologically suppressed HIV-infected individuals [147]. However, further investigation is required to elucidate how mtDNA activates inflammation during the development and progression of physical frailty and cognitive decline.

**Table 1:** The potential biomarkers between physical frailty and cognitive decline.

Physiological links	Potential biomarkers
Chronic inflammation	IL-6, IL-1β, IL-12, TNF-α, CRP, IL-10, IL-4, IL-13, IL-1Ra, sTNFR1
Impaired HPA stress response	DHEA-S, GH, Cortisol, testosterone
Imbalanced energy metabolism	IGF-1, SIRT1, Ghrelin
Endocrine dysregulation	IR, vaspin, adiponectin, leptin
Mitochondrial dysfunction	mtDNA
Oxidative stress	ROS
Genomic markers	*APOEε4, APOEε3, IL-6 rs1800796*, *TNF rs1800629*, *IL-18 rs360722*, *IL1-beta rs16944*, *COMT rs4680, COMT rs4646316*, BDNF, *CRP rs1205, IL-10 1082CC, IL-1α rs1800587, IL-1β rs1143634*
Metabolomic markers	LPC 18:2, LPC 18:1

Abbreviations: IL, interleukin; TNF-α, tissue necrosis factor alpha; CRP, C-reactive protein; sTNFR1, Soluble TNF receptor 1; HPA, hypothalamic-pituitary axis; DHEA-S, dehydroepiandrosterone sulfate; GH, growth hormone; IGF-1, insulin-like growth factor -1; SIRT1, Silent mating-type information regulation 2 homolog 1; IR, Insulin resistance; ROS, reactive oxygen species; mtDNA, mitochondrial DNA; ApoE, Apolipoprotein E; BDNF, Brain-derived neurotrophic factor; LPC, lysophosphatidylcholine.

## Oxidative stress

Frailty and cognitive decline are associated with oxidative stress (OS). OS was associated with accelerated aging, normal brain aging, and neurodegenerative diseases [148]. Oxidative damage accumulated with age and impaired cellular and organ function [149]. ROS contributed to skeletal muscle damage [150,151]. A recent review showed that frailty was associated with higher OS [152]. In the process of frailty, attenuated response of skeletal muscle to an increase in ROS levels contributed to a loss of ROS homeostasis and increased oxidative damage and age-related dysfunction in skeletal muscle [153]. Recently, Viña proposed a free-radical theory of frailty, postulating that oxidative damage is associated with frailty, but not with chronological age itself; their research on animals revealed that overexpression of antioxidant enzymes could delay the onset of frailty [154].

OS is associated with cognitive decline [155]. Chronic inflammation possibly alters immunological responses in the brain and further enhanced damage progression [156]. A pro-inflammatory environment with increased OS leads to endothelial dysfunction, which links cognitive impairment and frailty [148]. Thus, OS may serve as a common biological pathway that explains how physical frailty and cognitive decline are interrelated.

## Genomic markers

Genetic background can interact with inflammation and other mechanisms involved in the process of physical frailty and cognitive decline. Apolipoprotein E (ApoE) was associated with lifespan and cognitive function [157]. Carriers of the *APOEε4* allele had reduced CRP levels [158,159], and the association between increased CRP level and better cognition was observed only in older patients without the *APOEε4* allele [160,161]. When the aMCI group was stratified by the *APOEε4* status, significant differences were found in the levels of IL-6 and IFN-γ between the low- and high-risk groups and the control group [162], suggesting that some genetic factors are important. The loss of the *APOEε4* allele may be a vulnerability factor that contributes to the adverse effects of HPA axis dysregulation on cognition and has been suggested as the main risk factor for late-onset AD, while the *APOEε3* allele was associated with a more adaptive HPA axis response [163].

Six genes were associated with frailty and cognitive decline in Sargent’s recent review [164]: *IL-6 rs1800796*, *TNF rs1800629*, *IL-18 rs360722*, *IL1-beta rs16944*, and *COMT rs4680* for cognitive decline and *COMT rs4646316* for frailty. Brain-derived neurotrophic factor (BDNF) is involved in neuronal survival/proliferation processes. Decreased BDNF levels were associated with cognitive impairment, AD [165] and frailty [166,167]. The inhibition or degradation of BDNF antisense RNA has been reported to upregulate *BDNF* mRNA, increase BDNF protein levels, and induce neuronal growth and differentiation [168]. Thus, the SNP associated with BDNF may be related to the decreased plasma BDNF levels in frail people [166]. *TNF rs1800629* and *CRP rs1205* have been found to be associated with frailty [169,170]. *IL-10 1082CC*, associated with high serum levels of IL-10, was over-represented in centenarians [171]. *IL-1α rs1800587* and *IL-1β rs1143634* were significantly associated with AD onset [172]. *IL-6* gene variation was not associated with increased serum IL-6 levels or frailty [173,174] and the *IL-6 rs1800795* gene was not associated with sporadic AD [175].

## Metabolomic markers

Metabolomic markers may also contribute to the link between physical frailty and cognitive decline. Dysregulation of lipid metabolisms, such as higher phosphatidylcholine (PC) and lysophosphatidylcholine (LPC) levels, play a prominent role in age-related diseases such as dementia [176,177]. Many important physiological and pathophysiological processes are regulated by lysophospholipids and LPC was involved in inflammation [178]. Low levels of LPC species, such as LPC 18:2 and LPC 18:1, were associated with inflammation [179], IR [179], and AD [176]. Recently, a longitudinal study found that lower levels of blood LPC 18:2 were an independent predictor of physical function decline in older adults [180].

## Conclusion

Available evidence of the physiological links between physical frailty and cognitive decline from the observational studies is limited. The above findings provided initial insight into the potential roles of chronic inflammation, impaired HPA stress response, imbalanced energy metabolism, mitochondrial dysfunction, oxidative stress, and neuroendocrine dysfunction in the etiology of physical frailty and cognitive decline (Table 1). This provides important clinical implications for the easier identification of strategic approaches delaying the progression and onset of physical frailty and cognitive decline as well as preventing disability in the older population. Reversible functional and cognitive declines as defined in the construct of reversible cognitive frailty may be a target for secondary prevention for functional and cognitive impairment (Fig. 1), future clinical trials on biomarker-positive reversible cognitive frailty might be a promising direction [15,181]. While many biomarkers across multiple physiological systems are strongly associated with physical frailty and cognitive decline, it is notable that some results tend to be inconsistent between different studies, which poses a challenge and urgent need for future work on the physiological changes and identification of biomarkers for cognitive frailty.
